# PA28γ, an Accomplice to Malignant Cancer

**DOI:** 10.3389/fonc.2020.584778

**Published:** 2020-10-30

**Authors:** Kexin Lei, Hetian Bai, Silu Sun, Chuan Xin, Jing Li, Qianming Chen

**Affiliations:** State Key Laboratory of Oral Diseases, National Clinical Research Center for Oral Diseases, Chinese Academy of Medical Sciences Research Unit of Oral Carcinogenesis and Management, West China Hospital of Stomatology, Sichuan University, Chengdu, China

**Keywords:** PA28γ, cell proliferation, apoptosis, prognosis, treatment

## Abstract

PA28γ is a nuclear activator of the 20S proteasome, which is involved in the regulation of several essential cellular processes and angiogenesis. Over the past 20 years, many amino acid sites and motifs have been proven to play important roles in the characteristic functions of PA28γ. The number of binding partners and validated cellular functions of PA28γ have increased, which has facilitated the clarification of its involvement in different biological events. PA28γ is involved in the progression of various diseases, and its aberrant overexpression in cancer is remarkable. Patients with low levels of PA28γ expression have a higher survival rate than those with high levels of PA28γ expression, as has been shown for a wide variety of tumors. The functions of PA28γ in cancer can be divided into five main categories: cell proliferation, cell apoptosis, metastasis and invasion, cell nuclear dynamics that have relevance to angiogenesis, and viral infection. In this review, we focus on the role of PA28γ in cancer, summarizing its aberrant expression, prooncogenic effects and underlying mechanisms in various cancers, and we highlight the possible cancer-related applications of PA28γ, such as its potential use in the diagnosis, targeted treatment and prognostic assessment of cancer.

## Introduction

The proteasome system is an indispensable protein degradation system that has the responsibility of degrading the majority of the proteins in eukaryotes (1, 2). It can selectively act on misfolded, damaged, degenerative and nonfunctional proteins and turn them into peptide fragments (3), thus it regulates various molecular processes (4). Usually, the proteasome system comprises four main factors, namely, the 20S core and three proteasome activators (PA700/19S, PA28/11S, and PA200) (5).

The PA28 family has three members—PA28α, PA28β, and PA28γ, and there are several differences among them. First, PA28α and PA28β are only found in vertebrates, while PA28γ has been found in both vertebrates and invertebrates and is more highly conserved (6). Second, PA28α and PA28β share 50% of their amino acid sequence, while PA28γ shares only 25% of the sequence with the other two members (7). Third, PA28α and PA28β exist in the cytoplasm in the form of a heteroheptamer, but PA28γ exists primarily in the nucleus in the form of a homoheptamer (8). In addition, characterization of genomic clones for copies of PA28α and PA28β are not clear whereas PA28γ is probably encoded by a single-copy gene (9). Another difference is that the expression levels of both PA28α and PA28β can be enhanced by interferon-γ (IFN-γ) induction, and they take part in the formation of the hybrid proteasome 19S-20S-11S while there’s no clear evidence that PA28γ can form hybrid proteasomes (5). In contrast, PA28γ is not sensitive to IFN-γ, and it binds to the 20S proteasome to initiate protein degradation in an ATP/ubiquitin-independent manner (8). Moreover, PA28γ promotes cell proliferation and anti-apoptosis and is closely associated with the occurrence and development of cancer, which implies that the PA28γ/20S proteasome complex may be a target of anticancer therapy.

Clearly, PA28γ is a proteasome activator with special characteristics, and here, we concisely demonstrate the origination of PA28γ considering its function and regulatory mechanisms.

## The Characteristics of PA28γ

**Figure 1:**
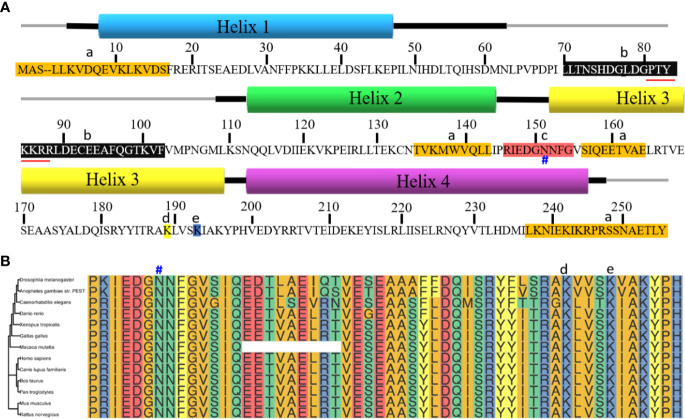
Sequence alignment of PA28γ. **(A)** PA28γ is composed of four helixes. The thick lines next to the helixes are the flanking sequences. a. Sequences highlighted in orange indicate those sequences that are different from the PA28α and PA28β. b. Homolog-specific insert is between helix 1 and helix 2 in the black background. It’s evolutionarily conserved and contains the nuclear localization sequence (marked with red underline), which binds the proteasome to other molecules. c. Activation loop is the sequence between helix 2 and helix 3 and is highlighted in red. N151Y (marked with #) blocks protein activation by binding to them, preventing them from binding to their normal counterparts. d.Lys188 (highlighted in yellow) enables PA28γ to stimulate all three proteasome catalytic sites. e. PA28γ (K195R) (highlighted in blue) can be acetylated mostly on its lysine 195 (Lys-195) residue by the CREB-binding protein (CBP), a modification that can be reversed by sirtuin 1 (SIRT1) in mammalian cells. Acetylation can influence the interaction between the monomers. **(B)** The evolutionary trees among multiple species with amino acids 145 to 199 of PA28γ. Multi-sequence alignment uses CLUSTAL 2.1 with default parameters, R packages ggtree and ggmsa were used for drawing graphics and evolutionary trees. N151 marked with #, Lys188 marked with d, and K195 marked with e above the corresponding amino acids.

PA28γ, also known as REGγ, 11Sγ, Ki antigen or PSME3, is encoded by the PSME3 gene (10). The human PA28γ gene is mapped to chromosome 17q12-21 (8). PA28γ is highly evolutionarily conserved in species such as tick, zebrafish, mouse, cattle and human beings (9, 11). The structure of PA28γ monomer is speculated to be composed of four helixes, and seven monomers will form a homoheptamer (12, 13). As speculated, there is a nine-residue sequence between helix 2 and helix 3, which is called activation loop. This sequence is the proteasome binding site and is vital for proteasome activation. A mutation in the activation loop N151Y, can suppress the action of the protein and inhibit proteasome activation by the nonmutated PA28γ. Another mutant, PA28γ (K195R), affects the acetylation of the lysine 195 (Lys195) residue by the cAMP response element binding protein (CBP), which can be restored by sirtuin 1 in mammals. Moreover, between helix 1 and helix 2, from Pro64 to Gly102, the sequence is called a homolog-specific insert. The insert can vary according to different homologs but has been highly conserved throughout evolution. The nuclear localization sequence (NLS) is in the insert. It may function by coupling proteasomes with nuclear components. Furthermore, in the sequence, the substitution of Lys188 with a negatively charged residue could enable PA28γ to activate three proteasome catalytic subunits (7) (**Figure 1A**). In addition, the evolutionary tree showed that the region containing multiple functional sites is very conserved among multiple species, Which region is amino acid sequence from 145 to 199 of PA28γ (**Figure 1B**).

Autoantibodies against PA28γ was first found in the serum of systemic lupus erythematosus (SLE) patients and was named the Ki antigen (14). However, the other biological functions of PA28γ are gradually eliminated. Early in 1989, Nikaido et al. deduced that PA28γ might be closely linked to cellular fission (15). Later, a study discovered that PA28γ-knockout mice showed considerable declines in their growth rates and body sizes compared with those of the control group. The embryonic fibroblasts of the knockout mice were hindered in the S stage of the cell cycle (13, 14). In conclusion, PA28γ is closely associated with cell division and DNA synthesis.

In recent years, studies have shown that transcript variants are closely correlated with the occurrence and development of tumors. So far, five transcript variants of PA28γ have been discovered. Among them, the newest to be identified was predicted and confirmed in our previous work. Compared with the other four isoforms of PA28γ, the fifth isoform missing sixth and fifth exons, it can encode the protein with a truncated form that retains the conserved residues (16). Further investigation is warranted to explore whether there are more transcript variants and their involvement in tumors formation and growth.

## The Regulation of PA28γ in Cancers

### Aberrant Expression of PA28γ in Cancers

Early in 2003, PA28γ was discovered to be abnormally overexpressed in papillary adenocarcinoma samples and anaplastic carcinoma, but its level was relatively low in multinodular goiters (17). Recently, several studies have evaluated the clinical significance of PA28γ through the use of large clinical cohorts. Debo Chen et al. found that the expression of PA28γ was closely connected with the differences in the TNM stages of colorectal cancer (CRC) tissues (18). For oral squamous cell carcinoma (OSCC), our team reached similar conclusions—the level of PA28γ was correlated with overall survival (OS) and disease-free survival (DSF) (19, 20).

**Figure 2:**
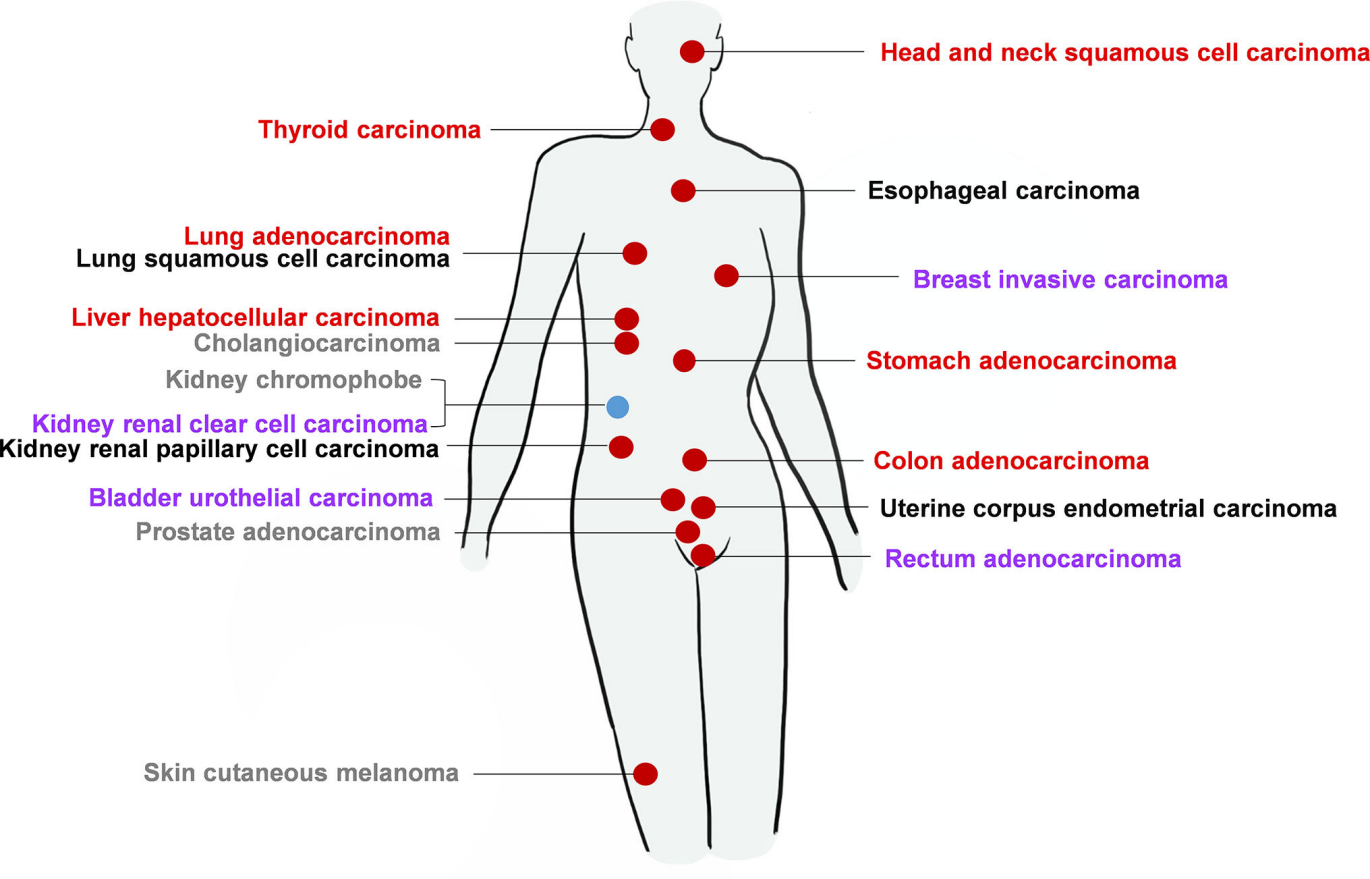
The expression of PA28γ and its relationship with cancer prognosis for a clinical cohort. According to the TCGA database, in most kinds of cancers, the level of PA28γ expression increases compared with that in normal tissues, which indicates that the level of PA28γ may be related to cell proliferation or malignancy. In some of the most common and serious cancers, such as lung carcinoma, breast invasive carcinoma, and head and neck squamous carcinoma, a higher level of PA28γ is associated with a shorter lifetime for the tumor patients. The red circle indicates increased levels of PA28γ in the tumor compared with that in normal tissue. The Blue circle indicates decreased levels of PA28γ in the tumor compared with that in normal tissue. The Red fonts indicate a negative trend in the expression of PA28γ and the lifetime of the tumor patients, according to the survival curve with P<0.05. Purple fonts indicate a negative trend in the expression of PA28γ and the lifetime of the tumor patients according to the survival curve, but P>0.05. Black fonts indicate no obvious negative trend in the expression of PA28γ and the lifetime of the tumor patients according to the survival curve with P>0.05. Gray fonts indicate that there is no current or relative evidence linking survival rate to the expression of PA28γ.

To eliminate the deviations in results caused by the heterogeneity of previous studies, we investigated the association between PA28γ and a variety of malignant tumors by analyzing PA28γ mRNA abundance in multiple human cancer tissues paired with normal tissues using the TCGA database. The results showed that most of the cancer samples had higher levels of PA28γ than the adjacent tissues except in kidney chromophobe and kidney renal clear cell carcinoma. Survival analysis of a wide variety of tumors revealed that the level of PA28γ was correlated with OS and DSF **(****Figure 2****)**. In conclusion, the expression of PA28γ is significantly increased in cancer tissues, and PA28γ expression in malignant tumors is closely related to their prognosis, suggesting that PA28γ plays an important role in the processes of cancer progression.

### Post-Translational Modification of PA28γ

PA28γ may undergo molecular modification when involved in various functions, typically acetylation. PA28γ can be acetylated at the K195 site. The cAMP response element binding protein (CBP), is responsible for modifying PA28γ by acetylation, and the deacetylase SIRT1 can block the CBP acetylation of PA28γ (21). Moreover, phosphorylation is another common modification. MEKK3, an upstream molecule of PA28γ, can increase the expression of PA28γ and directly modify PA28γ by initiating the phosphorylation of it. In addition, PA28γ can be SUMOylated both of *in vitro* and *in vivo* by SUMO-1, SUMO-2, and SUMO-3. The SUMO conjugation of PA28γ could cause increased stability of this proteasome activator and mediate its cytosolic translocation (22). Thus, these reports suggested that the post-translational modification of PA28γ is vital importance to its function.

## The Function and Mechanism of PA28γ in Cancer Progression

**Figure 3:**
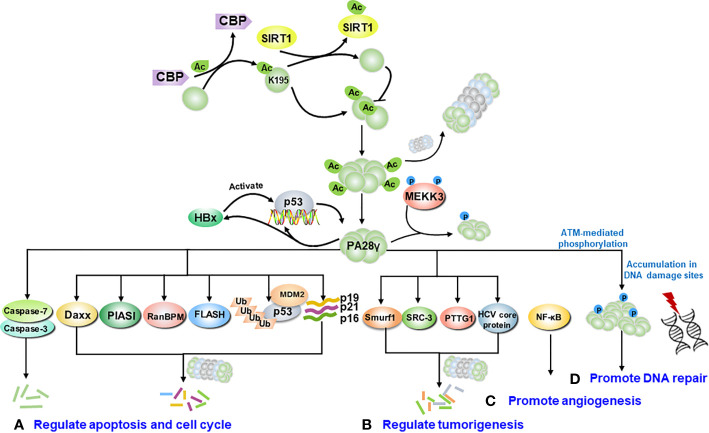
The molecular signaling pathway of PA28γ in cancer. After being modified, such as by acetylation or phosphorylation, PA28γ can interact with various molecules. **(A)** PA28γ regulates cell cycle and the apoptosis. PA28γ has been identified as substrate of caspase 3 and caspase 7, and it can degrade the apoptosis-related proteins FLASH, p16, p19, and p21. PA28γ is also involved in the MDM2-p53 interaction. **(B)** PA28γ can degrade tumor-related proteins such as Smurf1, SRC-3, PTTG1 and the HCV core protein, acting as a tumor suppressor. **(C)** PA28γ can promote the angiogenesis of tumors by activating NF-κB to promote the expression of IL-6 and CCL2 in oral squamous cell carcinoma (OSCC). **(D)** After ATM-mediated phosphorylation, PA28γ accumulates at DNA damage sites to promote DNA repair.

Hitherto, it was commonly recognized that PA28γ plays multifaceted roles in the cellular processes associated with malignant tumors. Closely related to its function, PA28γ can interact with many molecules. Here, we emphatically summarized the mechanisms into four categories such as regulate cell cycle and apoptosis, regulate tumorigenesis, promotion of angiogenesis, and promotion of DNA repair **(****Figure 3****)**.

### The Mechanism of PA28γ Regulate the Cell Cycle and Apoptosis

PA28γ could promotes tumor cell proliferation by affecting the cell cycle. Increasing evidence indicates that PA28γ is more highly expressed in tumor tissues than it is in normal adjacent tissues. It reportedly is highly expressed and plays an essential part in the proliferation of human laryngeal carcinoma cells (23), oral cancer cells (16), and prostate cancer cells (24), and leads to enhanced pathogenesis of the respective cancer. Cell nuclear dynamics, especially for DNA, constitute an essential stage during cell division. When PA28γ is knocked down, melanoma cell growth is inhibited and arrested in the G1 phase. Furthermore, the results showed that the DNA content in the G1 phase is increased but correspondingly is reduced in the G2 and S stages in PA28γ-knockdown melanoma cells compared to DNA content in the cells of the control group, as determined based on the average percentage of cells in the G1 phase (25). Moreover, scientists have discovered that PA28γ has the capability to degrade other intact tumor cell proliferation-related proteins, such as p16, p12, p19, p21 (26–30). Apoptosis of cancer cells is related to PA28γ, which primarily suppresses normal apoptosis. For instance, there are study demonstrated that after knocking down PA28γ, the cells of two different prostate cancer lineages showed a higher apoptosis ratio. The levels of a series of important molecules in cells were also significantly altered, such as p21, cyclin D1 and Bcl-2 (24). Moreover, In two different hepatocellular carcinoma cell lines, when PA28γ was downregulated by drug, the apoptosis rate significantly increased and the proportion of cells in the G2 phase dramatically decreased, which is found to be of potential in clinic (31).

**Figure 4:**
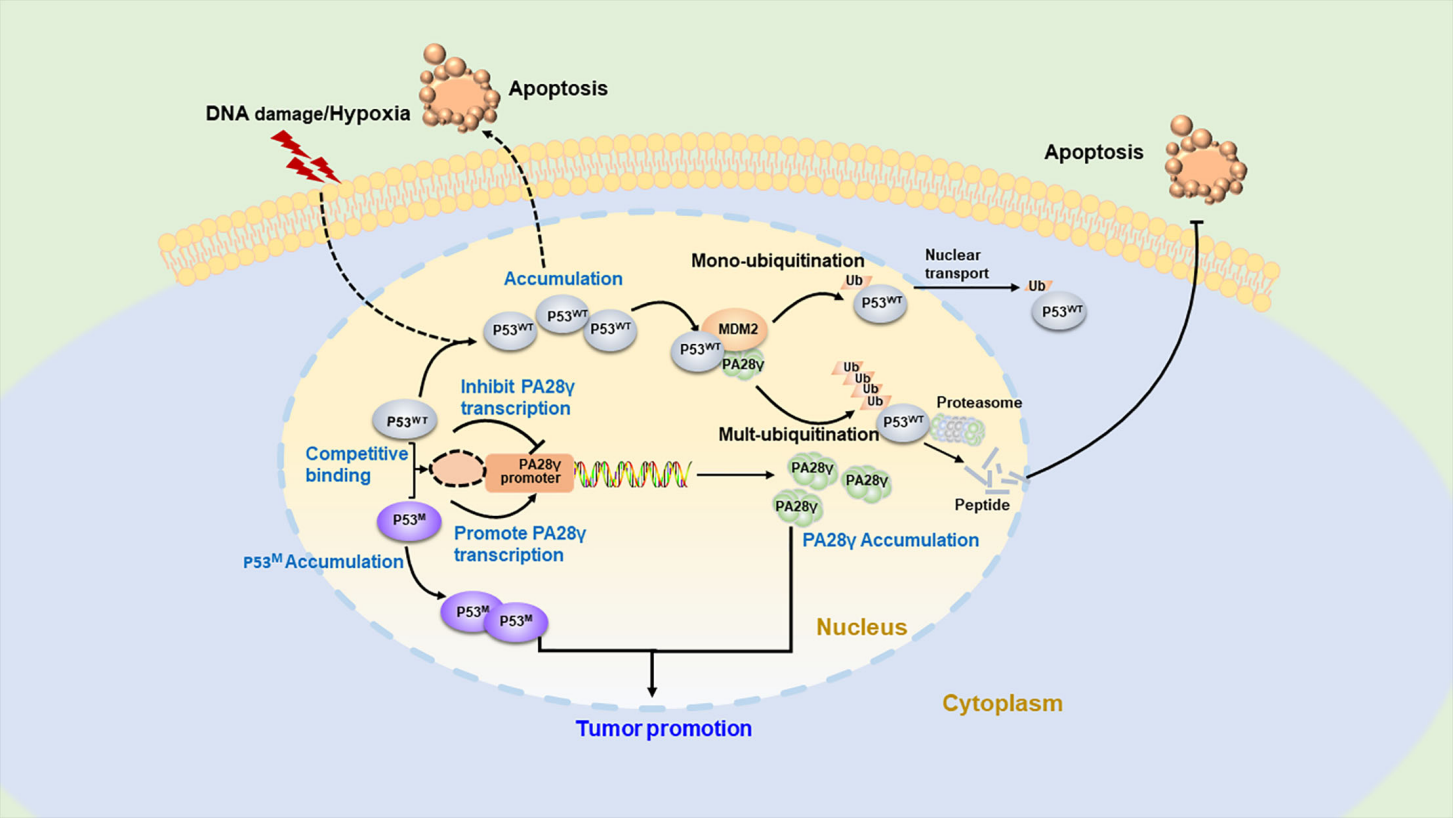
The regulatory feedback mechanism between PA28γ and P53. Overexpressed wild-type p53 can enhance the activity of the PA28 gene to upregulate PA28γ, while PA28γ can promote MDM2-mediated ubiquitination of p53, leading to the nucleation or degradation of p53. This is the negative feedback regulatory mechanism between p53 and PA28γ. In addition, mutant p53 can compete with wild-type p53 to bind to the site of PA28γ to promote PA28γ expression, thus inducing tumorigenesis.

Studies have indicated that PA28γ participates in the regulation mechanism of the apoptosis-related star molecules p53 (32) **(****Figure 4****)**. The p53 gene is the most frequently mutated anti-oncogene in human cancers, and most well-known functions are promoting cell cycle arrest and apoptosis (33, 34). MDM2 as an ubiquitin E3 ligase is the main negative regulator of p53 and affects the stability and function of p53 (35). It can induce the monoubiquitination of p53 and promote the nuclear export of the p53 protein. MDM2 also promotes the ubiquitination of p53, leading to the degradation of the p53 protein (36–38). Growing evidence suggests that PA28γ acts as a cofactor during the MDM2-p53 interaction, and the PSME3 is also the target gene of p53 (36). The accumulation of p53 can lead to the overexpression of PA28γ, which enhances the interaction between p53 and MDM2, promoting the degradation of p53. In this way, a negative feedback pathway is formed, which is conducive to ensuring the stability of p53 content. Cells deficient in PA28γ are sensitized to stress-induced apoptosis, suggesting that PA28γ plays a key role in the regulation of apoptosis, possibly by mediating the function of p53 *via* this MDM2-dependent pathway. In addition, scientists have also discovered that mutant p53 and wild-type p53 competitively bind to the sites of PSME3 to promote its accumulation along with the accumulation of mutant p53; consequently, tumor formation is promoted. p21, as a downstream gene of p53, is a broad-acting cyclin-dependent kinase inhibitor that can act as both a tumor suppressor and an oncogene. Therefore, it is assumed that PA28γ also plays a bidirectional role in tumor pathogenesis.

Taken together, the findings on the degradation of p53 and p21 shed light on the roles of PA28γ in the cell cycle and cancer. Moreover, PA28γ interactions with other proteins, including binding partners, that are associated with apoptosis, such as RanBPM, PIASI, FLASH, Daxx and SRC-3, MEKK3, p53 (39–41).

### The Mechanism of PA28γ Regulate Tumorigenesis

It is worth mentioning that, as we explain above, we have confirmed the high levels of PA28γ expression in malignant tissues and the inverse relationship between PA28γ content and survival time for cancer patients, and researchers have also verified that some PA28γ mechanisms promote tumorigenesis. However, in this regard, other evidence proves that PA28γ has two functions: it can degrade tumor-related proteins in some cases and/or play an antitumor role. SRC-3 is an oncogenic protein from the SRC family, which as a therapeutic target for breast cancer, prostate cancer and ovarian cancer (42, 43). In a study that undermined the traditional idea suggesting PA28γ can only degrade short peptides, PA28γ was first demonstrated to interact with SRC-3 and degrade this endogenous protein both *in vitro* and *in vivo* (44).

Furthermore, malignant metastasis and the invasion of tumors are induced when PA28γ is at abnormally high level. The motility and invasion capabilities of the cells decrease significantly after PA28γ is knocked down. High expression in breast cancer patients is closely associated with positive estrogen receptor alpha (ERα) status, leading to poor clinical prognosis (45). Correlation between nuclear PA28γ expression and invasion and relapse in hepatocellular carcinoma (HCC) (46).

Viral infection, especially infection with hepatitis C virus (HCV), is influenced by PA28γ. HCV is a major cause of chronic liver diseases, including steatosis, cirrhosis and hepatocellular carcinoma. The HCV core protein has long been proven able to bind with PA28γ not only in cell culture but also in the livers of HCV core transgenic mice, and overexpression of PA28γ leads proteolysis of HCV core protein (27). Moreover, knocking down PA28γ enhances the degradation of core proteins in ubiquitin-dependent way, impairs virus production, and leads to HCV core protein accumulation in the nucleus, thereby disrupting the development of both hepatic steatosis and HCC (47). The HCV core can induce transcriptional activation of PA28γ expression, in turn regulating its own protein level through a feedback loop that involves PA28γ in either ubiquitin-dependent or independent way (48). The association between PA28γ and human T-cell leukemia virus type 1 has also been proven (21).

### The Mechanism of PA28γ Promote Angiogenesis

PA28γ also has an impact on tumor angiogenesis. It contributes to tumor growth and is regarded as an important feature useful for evaluating the prognosis of cancer. Previously, PA28γ was reported as a novel angiogenic factor because it could ablation attenuates VEGF (vascular endothelial growth factor)-induced angiogenesis (49). Tumor-associated angiogenesis is of great importance in the metastasis and recurrence of tumors. In our previous study, we analyzed the expression of PA28γ in OSCC and found that the molecule could promote tube formation of Human Umbilical Vein Endothelial Cells (HUVECs) *in vitro* and accelerate tumor-induced angiogenesis in xenograft mouse models *in vivo* (50). We then found that the expression of PA28γ could promote the expression and secretion of IL-6 and CCL2 in OSCC cells by regulating the activation of NF-κB, thus promoting the angiogenesis of endothelial cells (50).

In addition, after phosphorylation by ATM, PA28γ is recruited to damage sites to boost DNA double-strand break repair *via* proteasome-mediated protein degradation. This ATM-PA28γ-proteasome axis is required for timely DNA repair (51).

## The Possible Application of PA28γ in Cancer Prognosis and Treatment

**Figure 5:**
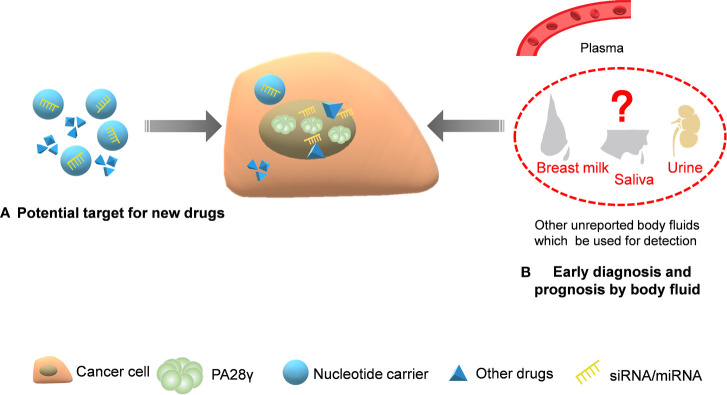
The application potential of PA28γ for cancer diagnosis and treatment. Because of its key indicative role in tumorigenesis and development, the potential clinical application value of PA28γ has been gradually explored. **(A)** Drugs and siRNA/miRNA that target PA28γ can affect signaling pathways and suppress tumor growth. **(B)** The content of PA28γ in body fluids, such as saliva, urine, and breast milk, may be of great importance in the early diagnosis and prognosis of cancer.

As demonstrated, PA28γ can affect different cell cycle inhibitors, is involved in important signaling pathways in tumor cells and is proven to play a part in the growth, metastasis, invasion, and angiogenesis of tumors. Consequently, PA28γ may hold the potential to act as a novel diagnostic biomarker, a therapy target and prognosis predictor **(****Figure 5****)**.

### The Application of PA28γ for Early Diagnosis

Currently, to develop minimally invasive or even noninvasive tools through blood or other body fluids for the early diagnosis of tumors, more and more researchers are focusing on discovering stable and reliable biomarkers. We believe that PA28γ has such potential for diagnostic applications. The upregulation of PA28γ in the serum of patients with colorectal cancer compared to that of healthy volunteers and patients with other bowel disease can be detected by a highly sensitive immunoassay, proving that PA28γ is able to act as a serum marker for CRC (18, 52). Theoretically, considering the functions of PA28γ in the cellular processes of cancer as described above, similar results may also be found for other kinds of carcinoma. Nevertheless, investigations on the application of PA28γ as an early-diagnostic biomarker are lacking. Additionally, before clinical application, the stability, sensitivity, and specificity of PA28γ will need to be examined carefully. Consequently, the clinical use of PA28γ requires more patience and hard work.

### The Application of PA28γ for Therapeutic Targeting

To date, several important intercellular molecules, complexes, and pathways have been reported to be influenced by the ubiquitin-independent and ATP-independent degradation induced by the PA28γ proteasome. Among those, most correlate closely with various tumor activities. Drugs targeting PA28γ are likely to act on the signaling pathway of PA28γ to affect the pathophysiology and development of malignant tumors. For example, researchers found abnormal downregulation of miR-7 in non-small-cell lung cancer (NSCLC) could promote cell proliferation, colony formation, and cell cycle progression, and cyclin D1 expression was lower when miR-7 levels were increased or PA28γ levels were decreased (12). In another study, after knocking down PA28γ, the level of p21 expression was increased, while the levels of cyclin D1 expression was decreased (24). These results may indicate that the miR-7/PA28γ/p21/cyclin D1 pathway may exist in cancer cells and that high levels of PA28γ may induce tumors to acquire a malignant phenotype. Taking advantage of the signaling pathway, the application of PA28γ in clinical treatment can be divided into two orientations, as explained below.

To support our hypothesis, we need to validate that PA28γ is at a low level in a patient, a state that can be the results of one of two functions: inhibiting its formation and or promoting its degradation. With regard to the first aspect, some investigators have already been able to inhibit PA28γ in ALVA41 prostate cancer cells, a natural amino monosaccharide, glucosamine, may downregulate PA28γ providing UDP-GlcNAc substrates for O-linked β-N-acetylglucosamine(O-GlcNAc) protein modification (53). A medicine named Tetrandrine could also downregulate PA28γ expression in different hepatocellular carcinoma cell lines. Tetrandrine can promote apoptosis and regulate the cell cycle by targeting PA28γ (31).

### The Application of PA28γ for Prognosis

As a biomarker, PA28γ plays not only a role in preoperative diagnosis but also as a prognostic indicator. High levels of PA28γ expression indicate metastasis and poor prognosis for patients with breast cancer. The disease-free and overall survivals indicators with low corresponding PA28γ expression are obviously more positive than they are for people with high PA28γ expression levels, demonstrating the possibility of utilizing PA28γ as a prognostic factor (45, 54). Similarly, PA28γ is a good predictor of the risk of death for patients with OSCC (19). However, currently, the potential of PA28γ as a biomarker is not particular popular, and its biomarker function has not been verified in some other tumors. More importantly, to date, there is no definitive criterion for abnormal expression, which requires numerous clinical trials in the future. Currently, the extraction and purification of PA28γ from serum and tissues are relatively widespread, which requires invasive methods for its detection, and we assume that it would be better if PA28γ could be tested in saliva or urine.

## Perspectives

Since PA28γ was discovered decades ago, a great deal of advances have been made in elucidating its characteristics and functions. Its novel expression patterns and diverse functions that promote pathological progress in cancers are relatively clear. The crucial roles in progress of carcinogens to the development of cancer have defined PA28γ as a promising therapeutic candidate. However, the exact role of PA28γ in cancer hasn’t yet been fully excavated. To date, the protein structure of PA28γ has not been resolved yet, and there are no commercial post-translational modification antibodies, such as phosphorylated antibodies, acetylated antibodies, and glycosylated antibodies. Limited by this, although some investigations have suggested that PA28γ can also participate in the tumor suppression process, the underlying mechanism still unknown, highlighting the need for further innovation in the field.

In terms of exploring the potential biomedical applications, accumulating evidence suggests that PA28γ holds great potential for use in early diagnosis, prognosis evaluation, and targeted therapy. However, as we describe above, the clinical utilization of PA28γ could be a new research direction for the many years to come. Thus far, numerous barriers need to be overcome, such as determining the means for accurate measurement, methods to prevent harm during assessments, and most importantly, a reliable criterion for determining effectiveness. All in all, sustained and systematic efforts (e.g. novel methods used in various pre-clinical cancer models) in the future are necessary for greater development and utilization of PA28γ.

## Data Availability Statement

The datasets generated and analyzed during the current study are available in the PubMed repository, www.ncbi.nlm.nih.gov/pubmed.

## Author Contributions

KL, HB, and QC wrote the manuscript and designed the figures. SS and CX collected the related references and edited the manuscript. JL provided guidance and revised this manuscript. All authors contributed to the article and approved the submitted version.

## Funding

This project was supported by the National Natural Science Foundation of China (numbers 81672675, 81872211, 81602375 and 81621062), the MoE III Project from China Higher Ed (grant number B14038), the CAMS Innovation Fund for Medical Sciences (CIFMS, 2019-I2M-5-004).

## Conflict of Interest

The authors declare that the research was conducted in the absence of any commercial or financial relationships that could be construed as a potential conflict of interest.

## Abbreviations

ATM, ataxia telangiectasia-mutated gene; ATP, adenosine triphosphate; Bcl-2, B-cell lymphoma-2; cAMP, cyclic adenosine monophosphate; CBP, cAMP response element binding protein; CCL2, chemokine (C-C motif) ligand 2; CRC, colorectal cancer; Daxx, death domain associate protein; DNA, deoxyribo nucleic acid; DSF, disease-free survival; ERα, estrogen receptor α; FLASH, FLICE-associated huge protein; Gly102, glycine 102; HCC, hepatocellular carcinoma; HCV, hepatitis C virus; HUVECs, human umbilical vein endothelial cells; IFN-γ, interferon-γ; IL-6, interleukin-6; Lys195, lysine 195; MDM2, murine double minute 2; MEKK3, mitogen-activated protein kinase/extracellular signal-regulated kinase kinase kinase 3; miR-7, micro RNA-7; NF-κB, nuclear factor kappa-B; NLS, nuclear localization sequence; NSCLC, non-small-cell lung cancer; OS, overall survival; OSCC, oral squamous cell carcinoma; O-GlcNAc, O-linked β-N-acetylglucosamine; PA200, proteasome activator 200; PA28, proteasome activator 28; PA28α, proteasome activator 28α; PA28β, proteasome activator 28β; PA28γ, proteasome activator 28γ; PA700, proteasome activator 700; PIASI, protein inhibitor of activated STAT, 1; PSME3, proteasome activator subunit 3; RanBPM, Ran binding protein in the microtubule-organizing center; SIRT1, Sirtuin-1; SLE, systemic lupus erythematosus; SRC-3, steroid receptor coactivator; TCGA, The Cancer Genome Atlas Program; UDP-GlcNAc, uridine 5’-[3-(acetamido-2-deoxy-α-D-glucopyranosyl) dihydrogen diphosphate]; VEGF, vascular endothelial growth factor.
